# DragonFly™ edge-to-edge repair of the systemic atrioventricular valve in a patient with congenitally corrected transposition of the great arteries: a case report

**DOI:** 10.1093/ehjcr/ytag238

**Published:** 2026-03-21

**Authors:** Xinping Lin, Boren Tan, Shih-Hsien Sung, Jian’an Wang, Xianbao Liu

**Affiliations:** Department of Cardiology, the Second Affiliated Hospital, School of Medicine, Zhejiang University, Jiefang Road 88, Hangzhou 310000, China; State Key Laboratory of Transvascular Implantation Devices, Qidi Road 456, Hangzhou 310000, China; Heart Regeneration and Repair Key Laboratory of Zhejiang Province, Jiefang Road 88, Hangzhou 310000, China; Transvascular Implantation Devices Research Institute, Qidi Road 456, Hangzhou 310000, China; Department of Cardiology, the Second Affiliated Hospital, School of Medicine, Zhejiang University, Jiefang Road 88, Hangzhou 310000, China; State Key Laboratory of Transvascular Implantation Devices, Qidi Road 456, Hangzhou 310000, China; Heart Regeneration and Repair Key Laboratory of Zhejiang Province, Jiefang Road 88, Hangzhou 310000, China; Transvascular Implantation Devices Research Institute, Qidi Road 456, Hangzhou 310000, China; Taipei Veterans General Hospital, No. 201, Sec. 2, Shipai Rd., Beitou District, Taipei City, Taiwan 11217, R.O.C; Department of Cardiology, the Second Affiliated Hospital, School of Medicine, Zhejiang University, Jiefang Road 88, Hangzhou 310000, China; State Key Laboratory of Transvascular Implantation Devices, Qidi Road 456, Hangzhou 310000, China; Heart Regeneration and Repair Key Laboratory of Zhejiang Province, Jiefang Road 88, Hangzhou 310000, China; Transvascular Implantation Devices Research Institute, Qidi Road 456, Hangzhou 310000, China; Binjiang Institute of Zhejiang University, Jucai Road 239, Hangzhou 310000, China; Department of Cardiology, the Second Affiliated Hospital, School of Medicine, Zhejiang University, Jiefang Road 88, Hangzhou 310000, China; State Key Laboratory of Transvascular Implantation Devices, Qidi Road 456, Hangzhou 310000, China; Heart Regeneration and Repair Key Laboratory of Zhejiang Province, Jiefang Road 88, Hangzhou 310000, China; Transvascular Implantation Devices Research Institute, Qidi Road 456, Hangzhou 310000, China; Binjiang Institute of Zhejiang University, Jucai Road 239, Hangzhou 310000, China

**Keywords:** congenitally corrected transposition of the great arteries, DragonFly^TM^, Transcatheter edge-to-edge repair, Atrioventricular valve, Case report

## Abstract

**Background:**

Congenitally corrected transposition of the great arteries (ccTGA) features atrioventricular and ventriculo-arterial discordance. A substantial proportion of patients with ccTGA develop systemic right ventricular dysfunction and overload, leading to functional systemic atrioventricular valve (SAVV) regurgitation that markedly worsens clinical outcomes. Isolated SAVV interventions, specifically transcatheter edge-to-edge repair (TEER), have been scarcely reported.

**Case summary:**

An 84-year-old man with ccTGA was referred for treatment of severe systemic atrioventricular valve regurgitation. The patient had a history of atrial fibrillation and presented with progressive heart failure symptoms despite optimal guideline-directed medical therapy (GDMT). Owing to advanced age, multiple comorbidities, and a prohibitive surgical risk, the multidisciplinary Heart Team opted for transcatheter edge-to-edge repair using the DragonFly^TM^ system. Systemic atrioventricular valve repair was successfully performed with implantation of two clips under transoesophageal echocardiographic guidance. The postoperative course was uneventful, with significant reduction of regurgitation and improvement in function status during follow-up.

**Discussion:**

Our case demonstrates that transcatheter edge-to-edge repair using the DragonFly™ system may represent a feasible alternative for selected patients with ccTGA and functional systemic atrioventricular valve regurgitation who are at prohibitive surgical risk. Careful patient selection and meticulous procedural planning based on multimodality imaging are essential to achieve optimal outcomes in this complex population.

Learning points

- Systemic atrioventricular valve disease is rare in patients with ccTGA, particularly when driven by functional deterioration of the systemic right ventricle.
- TEER using the DragonFly™ system may be a feasible and safe therapeutic option for high-risk patients with systemic atrioventricular valve regurgitation.
- Multimodality imaging plays a critical role in patient evaluation, procedural planning, and intraprocedural guidance for TEER in complex cases.

## Introduction

Congenitally corrected transposition of the great arteries (ccTGA) is characterized by atrioventricular and ventriculo-arterial discordance, resulting in a physiologically ‘corrected’ circulation. In this setting, the morphologic right ventricle supports the systemic circulation, and the systemic atrioventricular valve (SAVV), typically a morphologic tricuspid valve, is prone to regurgitation due to intrinsic valve abnormalities and progressive annular dilatation.^1^ Severe SAVV regurgitation accelerates systemic right ventricle remodelling and heart failure and is a major determinant of long-term prognosis.^2^ Although surgical SAVV repair or replacement can be beneficial when performed before advanced systemic right ventricular dysfunction, operative risk may be prohibitive in elderly or high-risk patients. Transcatheter edge-to-edge repair (TEER) offers a less invasive alternative,^3,4^ but experience in ccTGA remains limited.^5,6^ We report the first elderly patient with ccTGA and severe functional SAVV regurgitation, treated by TEER using the DragonFly^TM^ system, emphasizing multimodality imaging for procedural planning and guidance.

## Summary figure

**Figure:**
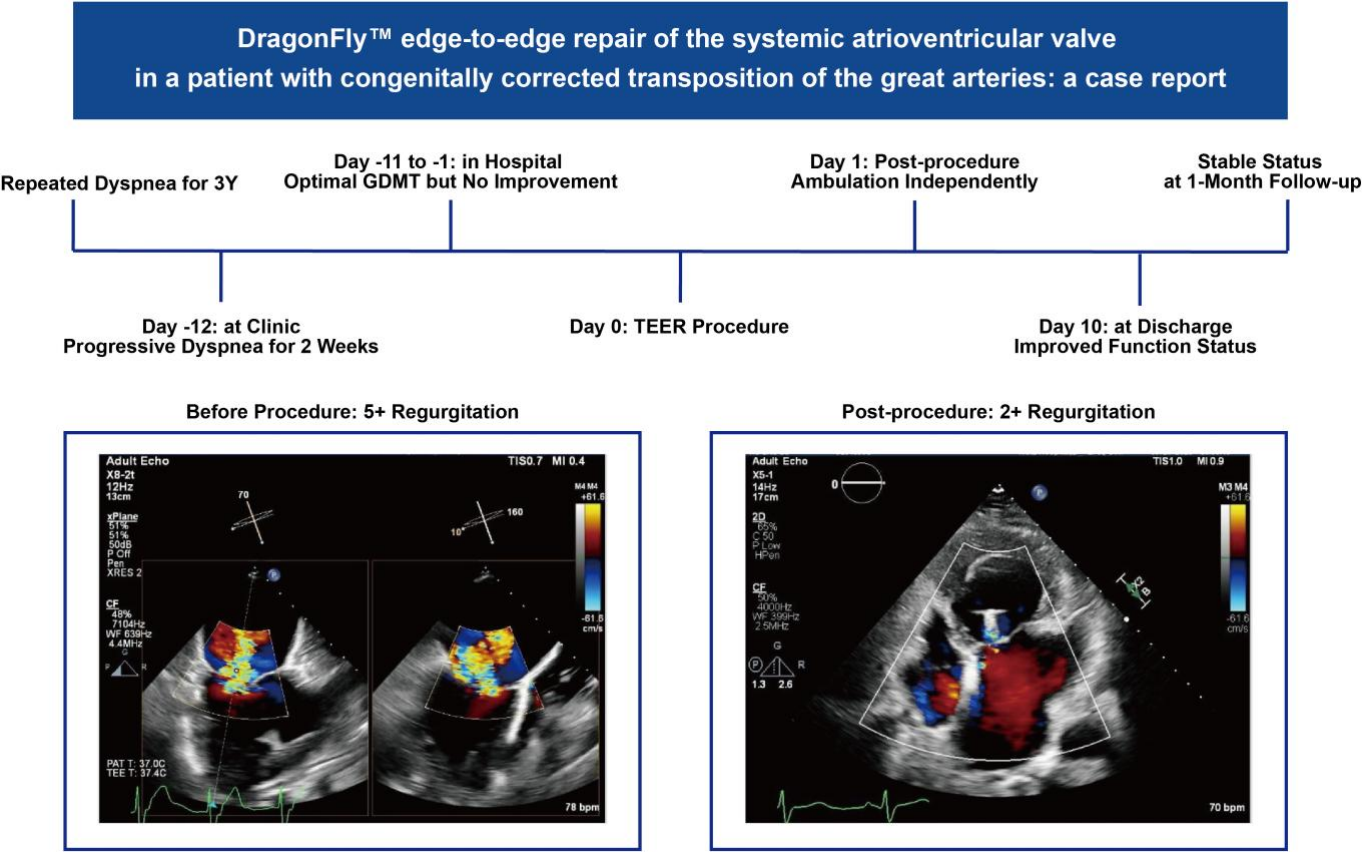


## Case presentation

An 84-year-old man was referred to our cardiology clinic due to progressive dyspnoea on exertion, accompanied by worsening bilateral leg oedema. His medical history included atrial fibrillation and chronic obstructive pulmonary disease. Physical examination revealed a grade 4/6 systolic murmur at the apex, diffuse lung crackles, and marked pitting oedema of both lower limbs. A transthoracic echocardiogram performed at a community clinic suggested severe atrioventricular valve regurgitation. Optimal medical therapy was initiated 1 month before this admission, including rivaroxaban 10 mg daily, dapagliflozin 10 mg daily, furosemide 20 mg daily, and spironolactone 20 mg daily, but symptoms remained refractory. Therefore, he was admitted for comprehensive assessment and management.

**Figure 1:**
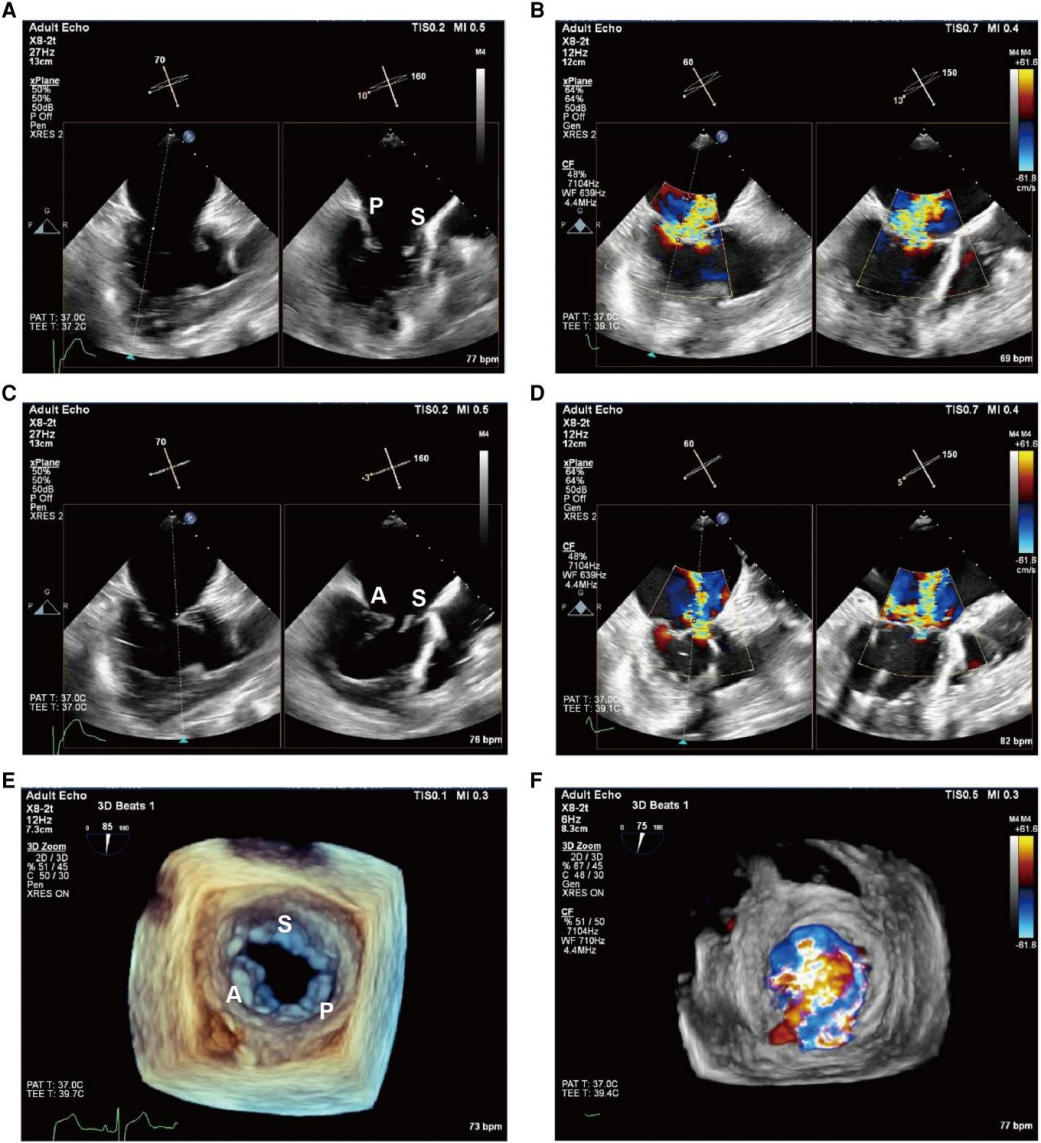
Transoesophageal echocardiography examination at mid-oesophageal. (*A–B*) Posterior–septal coaptation and significant regurgitation. (*C–D*) Anterior–septal coaptation and regurgitation. (*E–F*) Three-dimensional echocardiography demonstrating the leaflet anatomy, with regurgitation predominantly localized between the posterior and septal leaflets. A: anterior leaflet; P: posterior leaflet; S: septal leaflet.

**Figure 2:**
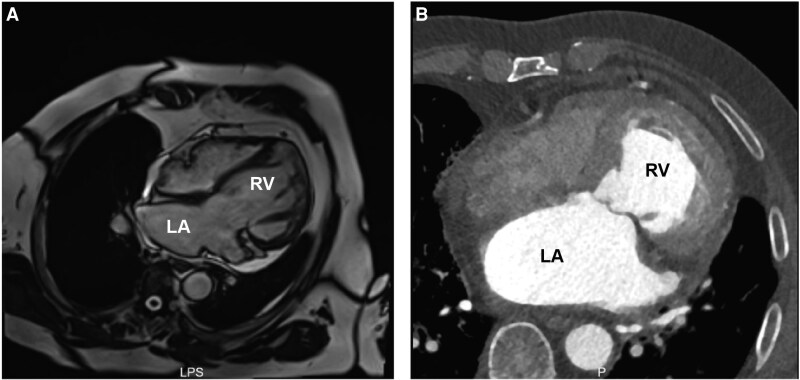
Four-chamber view on cardiac magnetic resonance (*A*) imaging and contrast-enhanced cardiac computed tomography (*B*). LA: left atrium; RV: right ventricle.

Upon admission, the patient was classified as New York Heart Association (NYHA) class IV (*Table 1*) and could not complete a 6-minute walk test because of severe exertional intolerance. Laboratory evaluation demonstrated markedly elevated B-type natriuretic peptide (BNP) of 2066.5 pg/mL and impaired renal function with a serum creatinine of 187.5 µmol/L. Transthoracic echocardiography confirmed ccTGA, severe SAVV regurgitation, and a dilated morphologic right ventricle (RV) with a systemic RV end-diastolic diameter of 6.65 cm. Transoesophageal echocardiography further characterized leaflet morphology and coaptation defects, demonstrating two prominent regurgitant jets at the posterior-septal junction region and anterior-septal region (*Figure 1*). Measured leaflet lengths were 1.71 cm (anterior), 2.39 cm (septal), and 1.82 cm (posterior). Cardiac computed tomography corroborated the anatomical features of ccTGA and a single coronary artery arising from the right sinus. Cardiac magnetic resonance (MRI) quantified systemic RV dysfunction with an ejection fraction of 40.71% and RV end-diastolic volume of 210.0 mL (*Figure 2*). Given persistent symptoms and advanced SAVV regurgitation with significant systemic RV remodelling despite medical therapy, intervention was indicated. However, a high Society of Thoracic Surgeons (STS) predicted risk score of 22.7% indicated prohibitive surgical risk for isolated SAVV replacement. Following multidisciplinary heart team discussion, TEER with the DragonFly^TM^ system was selected.

**Table 1:** Baseline, discharge and 1-month follow-up characteristics, echocardiographic and clinical parameters

	Baseline	Discharge	1-month Follow-up
Regurgitation Severity	5+	2+	2+
Mean transvalvular gradient, mmHg	2	2	2
IVSd, cm	0.95	1.08	1.11
RVIDd, cm	6.65	6.52	5.56
RVPWd, cm	1.04	0.96	1.03
RVEF, %	43.1	41.8	44.7
LAV, mL	159.3	148	91.6
LAVi, mL/m^2^	102.5	95.2	58.7
NYHA class	IV	II	II
BNP, pg/mL	2066.5	442.6	390.1
Creatinine, μmol/L	187.5	147.5	126.2

Abbreviations: BNP, brain natriuretic peptide; IVSd, interventricular septum diastole; LAV, left atrial volume; LAVi, left atrial volume index; NYHA, New York Heart Association; RVEF, right ventricular ejection fraction; RVIDd, right ventricular internal dimension diastole; RVPWd, right ventricular posterior wall dimension.

**Figure 3:**
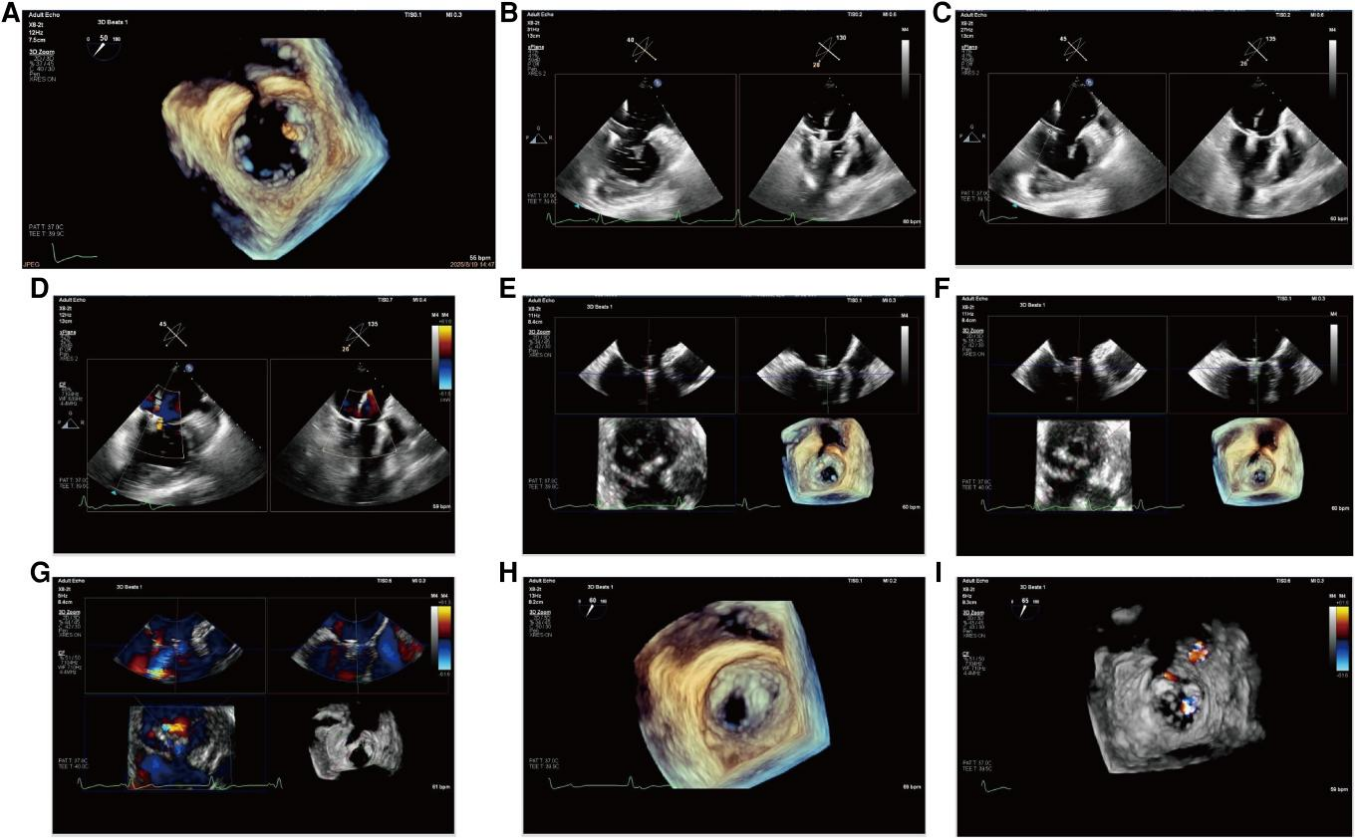
Stepwise transcatheter edge-to-edge repair of the systemic atrioventricular valve. (*A*) Orientation of the first clip perpendicular to the posterior–septal coaptation line under echocardiographic guidance. (*B*) Grasping of the posterior and septal leaflets. (*C–D*) Clip closure and stable leaflet capture leading to a marked reduction of posterior–septal regurgitation. (*E*) Positioning and orientation of the second clip perpendicular to the anterior–septal coaptation line, followed by leaflet grasping. (F–G) Closure of the second clip, with anterior–septal regurgitation reduced to trace severity. (H–I) Post-release three-dimensional echocardiography demonstrating stable tissue bridges created by both clips, with only mild to moderate (grade 1–2+) residual systemic atrioventricular valve regurgitation.

TEER was performed under transoesophageal echocardiographic guidance. After transseptal puncture (puncture height of 4.12 cm) from the morphologic right atrium (systemic venous atrium) to the morphologic left atrium (pulmonary venous atrium), a 24-F steerable guiding catheter was subsequently advanced over a stiff guidewire into the morphologic left atrium. A first DragonFly^TM^ 0609 clip was positioned perpendicular to the posterior-septal coaptation line and deployed with satisfactory leaflet grasping, resulting in a marked reduction in posterior-septal regurgitation (*Figure 3A–D*).

**Figure 4:**
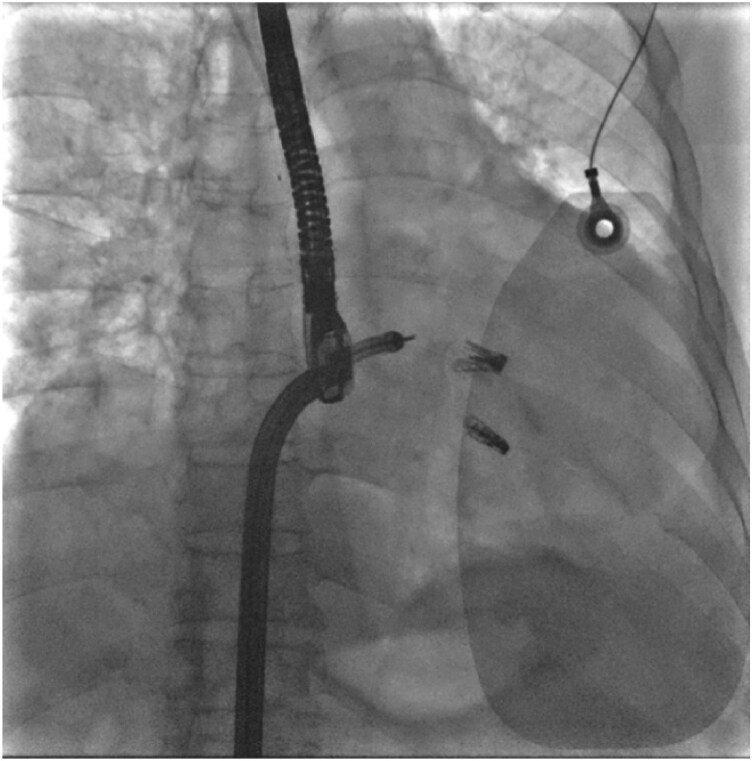
Fluoroscopic image demonstrating the two DragonFly™ clips implantation position.

Because severe regurgitation persisted from the anterior-septal commissure, a second DragonFly^TM^ 0612 clip was implanted perpendicular to the anterior-septal coaptation line. Gradual closure to an angle of 30° achieved trace residual regurgitation at anterior-septal coaptation (*Figure 3E–G*). Final assessment by transoesophageal echocardiography and digital subtraction angiography confirmed stable positioning of both device with only mild residual SAVV regurgitation (*Figure 3H* and *I*; *Figure 4*). Then, the steerable guiding catheter was withdrawn, venous access was closed, and the patient was transferred to the cardiac care unit for further management.

Pre-discharge transthoracic echocardiography demonstrated sustained reduction in SAVV regurgitation to grade 2+ with stable clip positions and no device-related complications (Summary Figure). At discharge, functional status improved to NYHA class II and BNP showed a downward trend (*Table 1*). At 1-month follow-up, repeat transthoracic echocardiography confirmed sustained device stability and persistent reduction of regurgitation. Systemic RV function improved, and the patient was classified as NYHA class II. No adverse events, rehospitalizations, or device-related complications were observed during follow-up.

## Discussion

In patients ccTGA, the morphologic RV as the systemic ventricle and is prone to progressive dilation and systolic dysfunction over time, eventually leading to heart failure.^2,7^ SAVV regurgitation commonly develops as systemic RV remodelling distorts the annulus and subvalvular apparatus, causing leaflet tethering and impaired coaptation. When atrial fibrillation coexists, annular remodelling and loss of coordinated atrial function may further accelerate functional SAVV regurgitation. Given the rarity and heterogeneity of ccTGA, reliably identifying patients who will rapidly progress to advanced RV failure and severe SAVV regurgitation remains challenging.^8^ In addition, the optimal treatment strategy for functional SAVV regurgitation is not well defined. Current evidence for transcatheter options is largely limited to case reports and small series^9–11^ suggesting feasibility of TEER using MitraClip or PASCAL in selected patients.

In this case, the DragonFly^TM^ system was firstly used for transcatheter repair of SAVV regurgitation in ccTGA patient. Multimodality imaging including TTE, TEE, cardiac CT and MRI was essential to delineate valvular anatomy, localized dominant jets, and guide procedural planning. Given that the dominant regurgitant jet originated from the posterior-septal commissure, a relatively high transseptal puncture was chosen to facilitate catheter steering, device alignment, and precise leaflet grasping. Clip selection was tailored to valve geometry that a long-arm, wide-span clip was selected for the larger coaptation gap at postero-septal region, followed by a short-arm, wide-span clip at the antero-septal region to maximize regurgitation reduction while preserving valve orifice area.

Clinically, the patient demonstrated early symptomatic improvement with sustained reduction in regurgitation. Serial assessment at baseline, pre-discharge, and 1-month follow-up showed progressive functional recovery accompanied by reverse remodelling of the systemic RV, supporting the effectiveness of TEER in this high-risk setting. Continued clinical and imaging surveillance is warranted to determine long-term outcomes and ventricular remodelling.

Some patients exhibit Ebstein-like morphology or leaflet prolapse, and standard transoesophageal views often require substantial modification. Even with three-dimensional imaging, identifying leaflet insertion and achieving reliable visualization may be difficult. In selected cases, complementary imaging modalities such as intra-cardiac echocardiography may provide real-time guidance for device positioning and leaflet grasping. Given the limited number of reported cases, larger studies and registries are needed to clarify patient selection, procedural strategy, and long-term outcomes.

To our knowledge, this is the first report describing DragonFly^TM^ TEER system for the treatment of the SAVV regurgitation in a patient with ccTGA. This case supports the feasibility and safety of a transcatheter edge-to-edge approach in a rare, high-risk, and complex clinical setting and underscores the critical role of multimodality imaging, as well as a multidisciplinary Heart Team.

## Data Availability

The original contributions presented in the study are included in the article/Supplementary material. Further inquiries can be directed to the corresponding author.

## Lead author biography

Professor Xianbao Liu is an interventional cardiologist at the Second Affiliated Hospital of Zhejiang University, School of Medicine. His research interests include percutaneous treatment in structural heart disease.

## Notes

**Consent:** Written informed consent for publication of this case report and images was obtained from the patient in accordance with COPE guidelines.
